# 

**Published:** 1893-05

**Authors:** 


### CONTENTS:

Article I.—Gleanings from the Journals, and Comments, By Edwin D. Downs, D. D. S. - - - - - 1
" II.—The Teeth During Pregnancy. - - - - - 12
" III.—Porcelain Crown- and Bridge-Work. By Dr. E. Parmly Brown. - - - - - - 15
" IV.—A Study of the Diseases of the Peridental Membrane, having their Origin at or near the Gingival Margin. By H. A. Kelley, D. M. D. - - - - - 19
" V.—Bacteria. By Dr. N. S. Hoff. - - - - - 25
" VI.—About Oxiphosphate. - - - - - - 28
" VII.—The New Mesmerism. - - - - - - 31

### COMMUNICATED.

World's Columbian Dental Congress. - - - - - - 34
Annual Meeting of the Chicago Dental Society. - - - 34

### EDITORIAL.

Diseased Breath. - - - - - - - - - - 35
Extraordinary Surgery. - - - - - - - - 37
Lockjaw. - - - - - - - - - - - 40
Prof. Chapin A. Harris. - - - - - - - - 41

### MONTHLY SUMMARY.

The Dentists at a Disadvantage. - - - - - - - 43
Diffuse Cellulitis of the Neck Due to Carious Teeth. - - - 45
Extract from a Discussion on Anchorage of Gold Fillings. - - 45
Articulating Lower Partials. - - - - - - - 46
Making Metal Dies from Plaster Molds. - - - - - 47
Welding Gold Crowns. - - - - - - - - - 47
Deaths Under Anæsthetics. - - - - - - - - 48
What to do in Chloroform Syncope. - - - - - - 48

